# Health-related quality of life in Italian children and adolescents with congenital heart diseases

**DOI:** 10.1186/s12872-022-02611-y

**Published:** 2022-04-15

**Authors:** Giulia Amodeo, Benedetta Ragni, Giulio Calcagni, Simone Piga, Salvatore Giannico, Marie Laure Yammine, Fabrizio Drago, Marta Luisa Ciofi degli Atti, Angela Rossi, Simona De Stasio, Teresa Grimaldi Capitello

**Affiliations:** 1 Unit of Clinical Psychology, Department of Neuroscience, Bambino Gesù Children’s Hospital IRCCS, Rome, Italy; 2 Department of Human Studies, LUMSA University, Rome, Italy; 3 Department of Pediatric Cardiology and Cardiac Surgery, Bambino Gesù Children Hospital IRCCS, Rome, Italy; 4 The European Reference Network for Rare, Low Prevalence and Complex Diseases of the Heart - ERN GUARD-Heart, Amsterdam, The Netherlands; 5 Clinical Epidemiology Unit, Bambino Gesù Children’s Hospital, Rome, Italy

**Keywords:** Health-related quality of life, Quality of life, Pediatric heart diseases, Congenital heart diseases

## Abstract

**Background:**

Congenital heart disease (CHD) is the most common congenital anomaly at birth, affecting approximately 1% of live births. In recent decades great medical and surgical advances have significantly increased life expectancy, shifting healthcare professionals' and researchers’ interests in patients' Quality of Life (QoL). The main aims of our study were to evaluate generic and condition-specific QoL in a group of Italian children and adolescents with CHD and their parents and examine the level of agreement and directional disagreement between child/adolescent and parents reports on generic and condition-specific QoL.

**Methods:**

A cross-sectional study was designed with CHD children and adolescents and their parents referred to the Cardiology Department of “Bambino Gesù” Children’s Hospital. The PedsQL scale was used, including generic (PedsQL 4.0) and cardiac-specific modules (PedsQL 3.0) were administered to patients and caregivers. A Kruskal–Wallis test was used to compare generic and cardiac module scores between patients with different ages, CHD diagnoses, and between patients who underwent surgery interventions and/or are currently taking cardiac medications.

**Results:**

498 families were enrolled in this study. On average, patients reported a good level of generic and condition-specific QoL, as well as their mothers and fathers. Children aged between 5–7 years old reported lower generic and cardiac-specific total QoL levels than children aged 8–12 years and adolescents (13–18 years). With regard to the agreement, patient-parent agreement on condition-specific QoL ranged from 25 to 75% while on generic QoL, it ranged from 19 to 76%. The highest percentage of disagreement between parents and children was found in patients aged 5–7 years old, both for condition-specific and generic QoL rates.

**Conclusions:**

Our study contributed to the growing body of knowledge on QoL in CHD, emphasizing the need for these families to receive support from multidisciplinary standardized care, including psychological consultations and support.

**Supplementary Information:**

The online version contains supplementary material available at 10.1186/s12872-022-02611-y.

## Background

Congenital heart disease (CHD) is the most common congenital anomaly at birth, affecting approximately 1% of live births [1]. In Italy, the prevalence of CHD is around 7 per 1000 births [2]. In the past two decades great medical and surgical advances have significantly increased life expectancy [3, 4] and the probability of survival beyond the first year of life has increased to 90% among individuals born with CHD [5, 6]. Thus, most children and adolescents with CHD are expected to reach adulthood [7, 8]. For this reason, there is a growing interest among healthcare professionals and researchers in patient-related outcomes, such as health-related quality of life (QoL) [9].

### The QoL of pediatric CHD patients

As a result of their condition and of the special medical care that they require, CHD patients often have behavioural, emotional, and cognitive needs that may impact their own and their families’ QoL [5, 9–11]. Previous studies [12–15] have shown that children and adolescents with CHD perceived lower levels of QoL than their healthy peers and had an increased incidence of mood disorders, delays in cognitive development, below average school performance, and poor social interactions [10, 13, 16, 17], especially during the school-aged period. The transition from home to school requires developing new skills, including physical activity, socialization, autonomy, and self-confidence, as well as new challenges for both children with CHD and their parents [11]. Nevertheless, while the existing correlation between QoL and the severity of CHD has been demonstrated when considering the physical dimension of QoL [8, 18, 19], many patients develop a process of resilience and sense of coherence, and therefore may perceive good QoL, particularly in the psychosocial dimensions [20, 21]. For this reason, depending on which aspect of QoL is taken into account (the physical or psychosocial domain), some studies have reported a better perceived QoL in pediatric CHD patients than in the general population [22], while other studies, focusing on the impact of the CHD severity on patients’ QoL, have reported lower levels of perceived QoL [12, 15, 20, 23].

Considering that no previous studies have investigated the QoL of Italian children with CHD and adolescents, the first aim of the current study was to evaluate the generic and condition-specific QoL of pediatric CHD patients (reported both by patients and their parents) referred to our institution by examining potential differences in QoL levels between ages (2-4y, 5-7y, 8-12y and 13-18y), between CHD diagnoses, and between patients who underwent surgical interventions and/or are taking cardiac medications at present, to inform clinicians on patients’ QoL.

### The role of parent proxy-reports

Even if patient self-reports should be preferred in clinical practice as the standard for evaluating their perceived QoL, there may be several situations in which parent proxy-reports are required. For example, when the children are too young, ill, fatigued, or cognitively impaired to complete the questionnaires. To date, while parental proxy-reports have been widely validated in the literature [24, 25], there is no consensus regarding the direction of the agreement between patients’ and parents’ perceptions of QoL in the CHD pediatric population. Studies assessing the QoL of pediatric cardiac patients have indeed indicated both agreement [26] and discordance [15, 27] between self-reports and parent proxy reports [28]. According to the literature, parental reports could provide important complementary information about the QoL of children [24, 25, 29]. Moreover, understanding the similarities and differences between parental and children reports is important considering that parental perceptions of the child’s psychosocial health could influence children’s access to health care [25]. For these reasons, the second aim of this study was to examine the level of agreement and directional disagreement between child/adolescent and parents reports on generic and condition-specific QoL.

To the best of our knowledge, this is the first study describing the QoL of Italian CHD pediatric patients and directional disagreement between parents and children with CHD and adolescents, including both mothers’ and fathers’ perspectives.

## Methods

The current study represents the first step of a larger research project aiming at analysing the QoL and psychological adjustment of Italian CHD patients and their families referred to the Cardiology Department of “Bambino Gesù” Children’s Hospital, a tertiary pediatric hospital located in Rome, Italy. Specifically, in this cross-sectional study, children, adolescents, and their parents were recruited from January to December 2018 at our institution during the cardiological Day Hospital (DH). Cardiological DH is a follow-up appointment offered to all patients with CHD seen at our institution. It is placed in the setting of a multidisciplinary standardized follow-up care. During DH appointments, children and adolescents undergo a comprehensive evaluation by different physicians, including pediatric cardiologists, surgeons, and psychologists, at preset time frames. All patients enrolled in our study were followed up in the cardiology outpatient clinic. Clinical examination, 12 lead ECG and 2D echocardiography were performed at regular time intervals according to their cardiac conditions. In addition, psychological tests and consultations were performed on the same day of the cardiac evaluation. At the beginning of the psychological evaluation the clinician explained the study procedure to the patients and their parents and asked for their consent to participate. Considering that all the patients were minors at the time of recruitment (under the age of 18 years), parents signed the consent for themselves and their children, but only after obtaining verbal consent from the patient. After obtaining informed consent, the parents and patients completed the QoL questionnaires independently in two separate rooms. Due to their developing writing and reading skills, preschool-aged children were assisted in filling out the questionnaires by the clinician. Specifically, the clinician helped the children read the questions and signed the answers indicated by the children, as in the Italian validation study [30]. Only after completing the QoL questionnaires, the clinician collected supplementary neuropsychological, emotional, or behavioral information during the consultation.

The inclusion criteria were: 1) patients were aged between 2 and 18 years, and 2) parents and patients were able to understand the questionnaires. Non-Italian speakers and children diagnosed with severe neurodevelopmental disorders and/or with a diagnosis of intellectual disability were excluded from the study. These children had previously received their diagnosis, before the cardiological DH, and the psychologist was able to identify them through the priority path reserved to them for the DH appointments.

Approval to conduct the study was given by the Ethics Committee of “Bambino Gesù” Children’s Hospital and all parents involved in the study signed written informed consent for them and their children to participate.

### Instruments

For QoL evaluation the *PedsQL Generic Core Scales 4.0 scale* [24, 31]-Italian version] and the *PedsQL 3.0 Cardiac Module* [32, 33]-Italian version] were used for patients and their caregivers. The generic module evaluates four domains of QoL: physical functioning (8 items), emotional functioning (5 items), social functioning (5 items) and school functioning (5 items). The cardiac-specific module evaluated seven different domains: cardiac symptoms (7 items), adherence to treatment (5 items—if the patient is on pharmacologic treatment), perceived physical appearance (3 items); anxiety towards treatment (4 items); cognitive status (5 items) and communication skills (3 items). For children between 2 and 4 years of age only caregivers answered the questionnaire. For self-reported QoL, the questionnaire was divided by age: preschool children (5–7 years of age), school children (8–12 years of age) and adolescents (13–18 years of age). The parent-proxy report is categorized equally for children between 2 and 18 years of age. Items are measured on a 5-point Likert scale from 0 = never to 4 = almost always. Scores are then transformed to a 0–100 scale where 0 = 100, 1 = 75, 2 = 50, 3 = 25 and 4 = 0. For children between 5 and 7 years-old the Likert scale is simplified to a 3-point scale as follows: 0 = never, 1 = sometimes and 2 = almost always [10, 13].

### Statistical analyses

Model assumptions of normality were checked before statistical analyses exploring skewness (asymmetry) and kurtosis (kurtosis) of each variable (skewness and kurtosis cut-off points were set to [− 2; + 2] [34]). Descriptive statistics for all the studied variables were reported in terms of median and interquartile range for continuous variables (scores for general and cardiac-specific QoL questionnaires) and of frequencies for categorical variables. Considering the non-normality of the distribution of the studied variables, nonparametric models were used. Specifically, a *Kruskal–Wallis* test was used to compare scores for generic and cardiac modules between patients of different ages (2–4 y, 5–7 y, 8–12 y and 13–18 y), between CHD diagnoses, and between patients who underwent surgical interventions and/or are taking cardiac medications at present. For each *Kruskal–Wallis* test performed, follow-up analyses were conducted (pairwise comparisons in SPSS) to determine which comparisons were significant between groups [35].

To be consistent with the aim of obtaining a comprehensive understanding of patients’ QoL and to allow clinicians to understand the perception of both the patients and their parents, the comparisons were tested and reported for all patients, mothers and fathers.

With regard to CHD diagnoses, patients were classified into six different categories, according to the congenital heart disease they had and the surgical treatment they had previously undergone. These 6 categories were: *Aortopathies* (Ao), which included all patients with aortic disease from the aortic valvular lesion to the aortic arch anomalies (i.e. aortic coartaction); *Tetralogy of Fallot* (ToF), independent from the surgical approach they had had (transannular or infundibular patch); *Univentricular hear*t (UVH), which included all patients who have treated with Glenn or Fontan palliation according to the UVH physiology they had; *Right ventricular-pulmonary artery conduit* (RV-PA conduit), which included all patients requiring a connection from RV to PA by conduit instead of the natural RV outflow tract; *Transposition to the Great Artery* (TGA), which included all patients with atrioventricular concordance and ventricular artery discordance, treated with arterial switch operation; and *Other Congenital Heart disease* (oCHD), which included a miscellaneous of diseases not considered above.

Finally, regarding the agreement and directional disagreement between child/adolescent and parent reports on generic and condition-specific, we calculated parent–child-disagreement as absolute and directional discrepancies [29, 36, 37]. Directional discrepancies were categorized into three groups (“parent-report < child-report”, “agreement”, and “parent-report > child-report”) based on the threshold for minimally important differences (MIDs) in quality of life. A previous meta-analysis by Norman et al. [38] showed that the threshold of discrimination for changes in QoL in patients with chronic health conditions appears to be approximately half a standard deviation (SD). Hence, we used this threshold of half an SD to evaluate essential differences between child- and parent-reports of QoL. All statistical analyses were performed using SPSS (Version 24.0. Armonk, NY: IBM Corp).

## Results

A total of 578 families were admitted to the cardiologic DH when the psychologist responsible for this study was present. Twenty families were excluded because they were non-Italian speakers, and 50 families were excluded due to their children's diagnosis of severe neurodevelopmental disorders and/or intellectual disabilities. Ten families refused to participate. Finally, 498 families agreed to participate and were enrolled. No significant differences emerged between the families who refused to participate and the families enrolled. Specifically, our sample was composed of 429 mothers (M_age_ = 46.15, SD = 5.30, range = 34–62), 323 fathers (M_age_ = 46.17, SD = 5.89, range = 30–62), 78 children aged 5–7 years old (M_age_ = 6.14, SD = 0.81), 178 children aged 8–12 years old (M_age_ = 10.35, SD = 1.35), and 195 adolescents aged 13–18 years old (M_age_ = 15.17, SD = 1.44). Patients reported an average total generic QoL of 78.19 (SD = 12.63) and an average total condition-specific QoL of 78.82 (SD = 11.66). Mothers reported an average total patient-generic QoL of 79.22 (SD = 15.48) and an average total patient-condition-specific QoL of 73.17 (SD = 13.61). Fathers reported an average total patient-generic QoL of 82.17 (SD = 14.78) and an average total patient-condition-specific QoL of 76.51 (SD = 13.62). The sociodemographic and clinical characteristics of the sample are reported in Table 1.

**Table 1:** Sociodemographic and clinical characteristics of the sample

**Patients’ age n (%)**	
2–4 y	47 (9.4)
5–7 y	78 (15.7)
8–12 y	178 (35.7)
13–18 y	195 (39.2)
**Patients’ gender n (%)**	
Male	296 (59.4)
Female	202 (40.6)
**CHD diagnosis n (%)**	
Ao	110 (22.3)
RV-PA conduit	52 (10.5)
TGA	62 (12.6)
ToF	74 (15)
UVH	63 (12.8)
oCHD	132 (26.8)
**Surgery/medications n (%)**	
No surgery or Medication	55 (11)
Surgery	278 (55.8)
Medications	27 (5.4)
Surgery + Medications	138 (27.7)
**Parental age M (SD; range)**	
Mothers	43 (6.25; 26–62)
Fathers	46 (5.84; 30–62)

*Ao* Aortopathies; *M* mean; *n* no. of patients; *oCHD* Other Congenital Heart disease; *RV-PA conduit* Right ventricular-pulmonary artery conduit; *SD* standard deviation; *TGA* Transposition to the Great Artery; *ToF* Tetralogy of Fallot; *UVH* Univentricular heart

### Differences in generic and cardiac-specific QoL according to sociodemographic and clinical factors

Results from the *Kruskal–Wallis* test showed potential differences in QoL levels between ages (Table 2), between CHD diagnoses (Table 3), and between patients who underwent surgical interventions and/or are taking cardiac medications at present (please see more information in the Additional file 1). These differences highlighted the importance of considering sociodemographic and clinical factors when assessing the QoL of CHD patients. The main results showed that children aged 5–7 years reported the lowest levels of both generic- (*H*(3) = 32.003, *p* = 0.000) and cardiac-specific (*H*(3) = 7.873, *p* = 0.020) total QoL. In contrast, adolescents reported a worse perceived physical appearance than children in the other groups (*H*(3) = 25.146, *p* = 0.000). With regard to parent-reports, parents of children aged 5–7 years reported more problems with communication skills than parents of patients in the other groups (mothers: *H*(3) = 24.121, *p* = 0.000; fathers: *H*(3) = 12.805, *p* = 0.005), and parents of adolescents, reported a worse perceived physical appearance than parents of children aged 2–4 (mothers: *H*(3) = 45.649, *p* = 0.000; fathers: *H*(3) = 32.857, *p* = 0.000) and lower adherence to treatment than parents of children aged 2–4 and 5–7 (mothers: *H*(3) = 11.906, *p* = 0.008; fathers: *H*(3) = 17.287, *p* = 0.001). When CHD diagnoses were considered, significant differences emerged between UVH patients and other patients. Indeed, both UVH patients and their parents reported more cardiac symptoms (*H*(5) = 12.146, *p* = 0.033), worse perceived physical appearance (*H*(5) = 19.856, *p* = 0.001), and lower physical functioning (*H*(5) = 15.243, *p* = 0.009) than other patients. Finally, patients who underwent surgery and took medications at present and their parents reported more cardiac symptoms than patients who did not undergo surgery and did not take medications. Moreover, they perceived lower levels of physical appearance, a lower total score at the generic PedsQL and lower school functioning levels than children who take medications only.

**Table 2:** Differences between ages

	2–4y	5–7y	8–12y	13–18y	*P* (Kruskal–Wallis)	Significant comparisons
	Median (IQR)	Median (IQR)	Median (IQR)	Median (IQR)		
**Patient report**						
*Peds cardio*		(n = 78)	(n = 178)	(n = 195)		
1.Cardiac symptoms	/NA	78.00 (27)	80.00 (21)	78.00 (18)	.070	
2.Adherence to treatment	/NA	100.00 (16)	90.00 (10)	95.00 (5)	.100	
3.Perceived physical appearance	/NA	**100.00** **(33)**	**100.00** **(27)**	**83.00** **(25)**	**.000**	13–18 < 5–7 13–18 < 8–12
4.Anxiety towards treatment	/NA	87.00 (50)	87.00 (40)	87.00 (19)	.158	
5.Cognitive status	/NA	**83.00** **(39)**	**77.50** **(30)**	80.00 (20)	**.024**	8–12 < 5–7
6.Communicative skills	/NA	**67.00** **(50)**	**83.00** **(33)**	**83.00** **(25)**	**.000**	5–7 < 8–12 5–7 < 13–18
7. Cardio total	/NA	**77.00** **(23)**	82.00 (16)	**81.00** **(14)**	**.020**	5–7 < 13–18
***Peds generic***		(n = 78)	(n = 177)	(n = 195)		
1.Physical functioning	/NA	**75.00** **(25)**	**79.50** **(22)**	**84.00** **(19)**	.**000**	5–7 < 8–12 5–7 < 13–18
2.Emotional functioning	/NA	70.00 (20)	75.00 (28)	75.00 (25)	.063	
3.Social functioning	/NA	**70.00** **(30)**	**87.50** **(28)**	**95.00** **(15)**	**.000**	5–7 < 8–12 5–7 < 13–18 8–12 < 13–18
4.School functioning	/NA	**80.00** **(30)**	**85.00** **(25)**	80.00 (25)	**.013**	5–7 < 8–12
5.Generic total	/NA	**74.00** **(13)**	**81.00** **(19)**	**83.00** **(15)**	**.000**	5–7 < 8–12 5–7 < 13–18
**Mother report**						
*Peds cardio*	(n = 44)	(n = 69)	(n = 161)	(n = 154)		
1.Cardiac symptoms	86.00 (24)	78.00 (34)	79.00 (33)	78.00 (18)	.218	
2.Adherence to treatment	**100.00** **(29)**	100.00 (8)	95.00 (15)	**90.00** **(15)**	**.008**	13–18 < 2–4
3.Perceived physical appearance	**100.00** **(17)**	**83.00** **(59)**	**75.00** **(42)**	**83.00** **(33)**	**.000**	2–4 > 5–7 2–4 > 8–12 2–4 > 13–18
4.Anxiety towards treatment	75.00 (94)	56.00 (75)	69.00 (75)	69.00 (55)	.903	
5.Cognitive status	**42.00** **(42)**	**55.00** **(48)**	**65.00** **(25)**	**75.00** **(35)**	**.000**	2–4 < 5–7 2–4 < 8–12 2–4 < 13–18 5–7 < 13–18 8–12 < 13–18
6.Communicative skills	**83.00** **(25)**	**67.00** **(34)**	**83.00** **(33)**	**75.00** **(58)**	**.000**	5–7 < 2–4 5–7 < 8–12 5–7 < 13–18
7.Cardio total	71.50 (32)	**68.00** **(23)**	75.00 (21)	**77.00** **(26)**	**.036**	5–7 < 13–18
***Peds generic***	(n = 45)	(n = 69)	(n = 161)	(n = 154)		
1.Physical functioning	91.00 (24)	91.00 (37)	91.00 (28)	85.50 (20)	.330	
2.Emotional functioning	75.00 (29)	75.00 (30)	75.00 (30)	75.00 (25)	.682	
3.Social functioning	95.00 (19)	90.00 (25)	90.00 (25)	92.50 (25)	.266	
4.School functioning	83.00 (40)	75.00 (20)	80.00 (30)	80.00 (30)	.130	
5.Generic total	86.00 (12)	81.00 (23)	83.00 (25)	81.00 (19)	.387	
**Father report**						
***Peds cardio***	(n = 30)	(n = 57)	(n = 117)	(n = 118)		
1.Cardiac symptoms	86.00 (20)	82.00 (27)	82.00 (25)	86.00 (18)	.537	
2.Adherence to treatment	100.00 (29)	**100.00** **(0)**	100.00 (10)	**92.50** **(20)**	**.001**	13–18 < 5–7
3.Perceived physical appearance	**100.00** **(17)**	**100.00** **(17)**	**83.00** **(34)**	**83.00** **(33)**	**.000**	8–12 < 5–7 8–12 < 2–4 13–18 < 2–4
4.Anxiety towards treatment	90.50 (97)	75.00 (75)	62.00 (75)	72.00 (50)	.259	
5.Cognitive status	**33.00** **(59)**	**60.00** **(43)**	**70.00** **(30)**	**80.00** **(40)**	**.000**	2–4 < 8–12 2–4 < 13–18 5–7 < 13–18 8–12 < 13–18
6.Communicative skills	92.00 (17)	**67.00** **(50)**	**92.00** **(42)**	83.00 (50)	**.005**	5–7 < 8–12
7.Cardio total	77.50 (18)	75.00 (20)	77.00 (19)	80.00 (21)	.110	
***Peds generic***	(n = 31)	(n = 56)	(n = 117)	(n = 119)		
1.Physical functioning	91.00 (18)	91.00 (24)	94.00 (122)	91.00 (16)	.456	
2.Emotional functioning	80.00 (24)	75.00 (25)	80.00 (30)	80.00 (28)	.288	
3.Social functioning	100.00 (10)	90.00 (20)	90.00 (25)	90.00 (20)	.231	
4.School functioning	79.00 (40)	85.00 (23)	80.00 (25)	85.00 (35)	.814	
5.Generic total	86.00 (15)	87.50 (18)	85.00 (24)	86.00 (17)	.432	

Bold are reported comparisons statistically significant

*IQR* Interquartile Range; *n* no. of patients

**Table 3:** Differences between CHD diagnoses

	oCHD	Ao	RV-PA conduit	TGA	ToF	UVH	*P*(Kruskal–Wallis)	Significant comparisons
	Median (IQR)	Median (IQR)	Median (IQR)	Median (IQR)	Median (IQR)	Median (IQR)		
**Patient report**								
***Peds cardio***	(n = 112)	(n = 101)	(n = 50)	(n = 60)	(n = 64)	(n = 61)		
1.Cardiac symptoms	**78** **(25)**	82 (13)	78 (34)	78 (17)	78 (18)	**75** **(22)**	**.033**	UVH < oCHD
2.Adherence to treatment	90 (14)	95 (10)	100 (8)	97.5 (10)	100 (14.5)	95 (5)	.060	
3.Perceived physical appearance	**100** **(13)**	**100** **(8)**	92 (17)	92 (33)	96 (25)	**83** **(42)**	**.001**	UVH < oCHD UVH < Ao
4.Anxiety towards treatment	87 (44)	87 (50)	87 (19)	87 (23.5)	87 (40.5)	87 (31)	.925	
5.Cognitive status	80 (10)	75 (20)	77.5 (33)	80 23.7)	75 (30)	75 (30)	.461	
6.Communicative skills	83 (33)	83 (46)	83 (55)	83 (31)	83 (33)	83 (42)	.849	
7.Cardio T OTAL	81.5 (15)	82 (16)	79 (25)	78.5 (11.75)	81.5 (15)	80 (17)	.739	
***Peds generic***	(n = 112)	(n = 101)	(n = 50)	(n = 60)	(n = 63)	(n = 61)		
1.Physical functioning	**84** **(33)**	81 (19)	**75** **(37)**	81 (20.25)	81 (21)	**75** **(25)**	**.009**	RV-PA < oCHD UVH < oCHD
2.Emotional functioning	80 (25)	75 (35)	70 (20)	77.5 (30)	70 (28.75)	75 (40)	.529	
3.Social functioning	90 (18)	90 (18)	90 (18)	85 (28.75)	90 (30)	85 (30)	.501	
4.School functioning	80 (10)	85 (33)	80 (13)	80 (25)	80 (35)	80 825)	.753	
5.Generic total	83.5 (20)	79 (23)	77 (19)	79.5 (13)	81 (23)	77 (24)	.088	
**Mother report**								
***Peds cardio***	(n = 114)	(n = 101)	(n = 43)	(n = 49)	(n = 64)	(n = 52)		
1.Cardiac symptoms	**82** **(38)**	**82** **(20)**	**82** **(32)**	**82** **(18)**	80.5 (21)	**71** **(21)**	**.001**	UVH < oCHD UVH < Ao UVH < RV-PA UVH < TGA
2.Adherence to treatment	95 (18)	95 (13)	90 (15)	92.5 (18.75)	97.5 (7.25)	95 (10)	.914	
3.Perceived physical appearance	**85** **(29)**	**87** **(30)**	92 (59)	83 (37.5)	83 (33)	**72.5** **(33)**	**.003**	UVH < oCHD UVH < Ao
4.Anxiety towards treatment	75 (72)	69 (69)	75 (54)	69 (44)	69 (43)	53 (69)	.815	
5.Cognitive status	60 (45)	65 (20)	70 (48)	55 (33.5)	60 (37.25)	67.5 (35)	.592	
6.Communicative skills	75 (34)	83 (33)	83 (67)	75 (34)	83 (33)	79 (67)	.405	
7.Cardio total	73 (27)	73 (21)	77 (30)	74 (18.5)	73 (19.5)	71 (22)	.850	
***Peds generic***	(n = 114)	(n = 101)	(n = 43)	(n = 49)	(n = 64)	(n = 53)		
1.Physical functioning	**91** **(33)**	**91** **(22)**	87 (43)	**91** **(22)**	84 (24.25)	**78** **(41)**	**.016**	UVH < Ao UVH < TGA UVH < oCHD
2.Emotional functioning	80 (45)	75 (25)	80 (50)	70 (25)	70 (28.75)	70 (30)	.360	
3.Social functioning	95 (33)	90 (23)	90 (23)	95 (22.5)	90 (30)	80 (40)	.122	
4.School functioning	90 (30)	80 (28)	85 (48)	75 (25)	75 (35)	75 (35)	.519	
5.Generic total	86 (36)	84 (24)	81 (33)	81 (18.5)	79.5 (26.5)	71 (32)	.091	
**Father report**								
***Peds cardio***	(n = 76)	(n = 65)	(n = 41)	(n = 46)	(n = 50)	(n = 43)		
1.Cardiac symptoms	**86** **(20)**	86 (20)	82 (41)	86 (15.7)	82 (18)	**75** **(22)**	**.010**	UVH < oCHD
2.Adherence to treatment	95 (18)	95 (14)	90 (13)	10 (20)	100 (0)	10 (10)	.426	
3.Perceived physical appearance	**100** **(21)**	**100** **(38)**	83 (38)	92 (25)	83 (25)	**75** **(33)**	**.000**	UVH < oCHD UVH < Ao
4.Anxiety towards treatment	75 (72)	75 (47)	75 (63)	75 (50)	75 (50)	56 (56)	.246	
5.Cognitive status	70 (35)	70 (20)	70 (28)	70 (35)	65 (32.5)	70 (40)	.654	
6.Communicative skills	92 (25)	87.5 (33)	83 (79)	79 (42)	92 (27)	83 (50)	.815	
7.Cardio total	78.5 (21)	76 (20)	76 (25)	77.5 (15.7)	77 (16)	76 (14)	.606	
***Peds generic***	(n = 77)	(n = 64)	(n = 41)	(n = 46)	(n = 50)	(n = 44)		
1.Physical functioning	94 (22)	**94** **(19)**	91 (60)	92.5 (22)	87 (25.75)	**82.5** **(25)**	**.009**	UVH < Ao
2.Emotional functioning	80 (40)	85 (35)	80 (35)	75 (25)	77.5 (12.5)	72.5 (35)	.172	
3.Social functioning	100 (28)	90 (20)	90 (28)	97.5 (20)	90 (20)	85 (45)	.060	
4.School functioning	85 (20)	85 (28)	80 (33)	80 (31.25)	80 (35)	85 (35)	.616	
5.Generic total	88 (24)	88 (23)	86 (36)	88 (17.25)	82 (24)	79.5 (30)	.063	

Bold are reported comparisons statistically significant

*Ao* Aortopathies; *IQR* Interquartile Range; *n* no. of patients; *oCHD* Other Congenital Heart disease; *RV-PA conduit* Right ventricular-pulmonary artery conduit; *TGA* Transposition to the Great Artery; *ToF* Tetralogy of Fallot; *UVH* Univentricular heart

### Agreement and directional disagreement between child/adolescent and parent reports on generic and cardiac-specific QoL

**Fig. 1:**
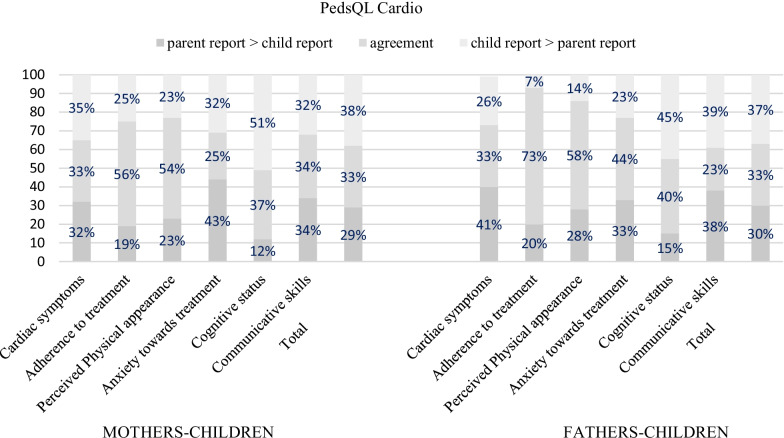
Agreement and directional disagreement between 5–7 y. 'patients and parents' reports

**Fig. 2:**
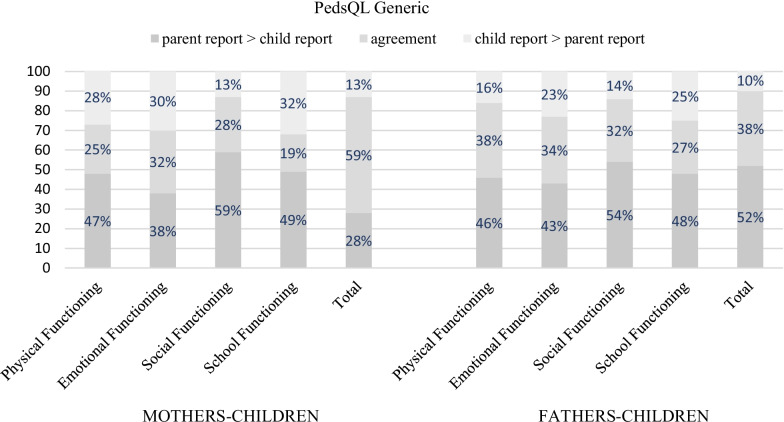
Agreement and directional disagreement between 5–7 y. 'patients and parents’ reports

The agreement between mothers and fathers on generic QoL varied from 47 to 86%, while on condition-specific, it varied QoL between 52 and 100% (detailed information is reported in Additional file 2). Patient-parent agreement on condition-specific QoL ranged from 25 to 75% while on generic QoL, it ranged from 19 to 76% (detailed information is reported in Additional file 3). The highest percentage of disagreement between parents and children was found in patients aged 5–7 years old, both for condition-specific (Fig. 1) and generic QoL (Fig. 2) rates. With regard to condition-specific QoL, mothers of 5–7 aged children underrated their children’s anxiety symptoms related to treatment (disagreement rate 43%; *e.g., I get scared when I am waiting to see the doctor, I get scared when I have to go to the doctor*). Moreover, children reported higher scores on cognitive problems (e.g., *It is hard for me to figure out what to do when something bothers me, I have trouble solving math problems, It is hard for me to pay attention to things*) than both their mothers (disagreement rate 51%) and their fathers (disagreement rate 45%). Finally, an agreement of 23% was found between patient and father reports in communication skills.

For generic QoL, both mothers and fathers showed a trend of underrating their children’s problems with generic physical, emotional, social and school functioning (disagreement rates from 38 to 59%).

## Discussion

According to Varni et al. [24] and Huang et al. [39], who identified cut-offs for patient-reported and parent-reported generic QoL, the majority of our patients (75%) and their parents (mothers: 61–80%; fathers: 74–84%) reported high levels of generic QoL, above the clinical cut-offs. These QoL levels may reflect the medical advances in screening and care for CHD in recent decades [40, 41]. Indeed, to date, over 90% of children with a CHD are expected to survive more than 30 years after the first cardiac surgery [3, 10, 40–42]. In addition to this, our patients and their parents are involved in a multidisciplinary standardized follow-up care. They are submitted to a comprehensive evaluation, including psychological consultations (providing support for patients and caregivers) that may contribute to enhancing their perceived QoL. According to these results, the literature has also shown that patients with congenital CHD are prone to cope with their health condition and develop adaptive skills earlier than their peers [21, 43, 44]. Indeed, according to previous studies, they might have learned early in their life how to develop a strong ‘‘sense of coherence’’ (understandability of the internal and external stimuli received during childhood; perception of the resources available to deal with stressful situations, such as living with heart disease; the ability of the individual to believe that his life has meaning, find motivation and assume the control over his life) and select the right coping strategies that may result in good QoL levels, especially in the psychological domains [11, 44]. Several studies, worldwide, have found good levels of QoL in paediatric CHD patients [4, 10, 11, 20, 22, 42, 45]. For example, Abassi et al. [11] examined a sample of 5- to 7-year-old French children and their parents; the mean total generic PedsQL scores from patient reports, mother reports, and father reports were 73.5, 76.1 and 79.2, respectively. Reiner et al. [22], in a sample of CHD patients from Germany aged 7–17 years, found a total generic QoL mean score of 78.6, and Moreno-Medina et al. [10] found at baseline a total score of 74.4 for the generic module and 79.6 for the cardiac module from a sample of 5–18-year-old CHD patients from Colombia; parents had a total score of 68.4 for the generic module and 73.6 for the cardiac module.

Nevertheless, the majority of these studies have mainly investigated the QoL of CHD patients through the PedsQL generic module, not including the cardiac-specific module. Moreover, they have focused only on specific age ranges and did not include both mothers and fathers in proxy-report evaluations. For these reasons, it is difficult to compare our results with this previous heterogeneous body of literature; moreover, no previous studies have been published on the QoL of Italian pediatric CHD patients. Our study has provided a comprehensive description of both generic and condition-specific QoL among Italian pediatric CHD patients at different ages (from 2 to 18 years), considering clinical factors such as CHD diagnosis and including both maternal and paternal reports. Our results can usefully inform clinical practice and guide clinicians’ efforts aimed at prevention and intervention.

### Differences in generic and cardiac-specific QoL related to age and clinical factors

With regard to the QoL levels of our patients in relation to their age and clinical factors, our results showed several differences between ages, CHD diagnoses, and between patients who underwent surgical interventions and/or are taking cardiac medications at present, highlighting the importance of considering sociodemographic and clinical factors when assessing the QoL of CHD patients. The main results showed that children aged 5–7 years reported the lowest generic- and cardiac-specific total QoL levels. During the transition from home to preschool or primary school, children should acquire skills that promote emotion recognition and regulation, empathy for others, problem-solving, and positive social interactions [46]. Furthermore, they are expected to show increasing independence and separation from their caregivers as they adjust and hone their social interactions with peers and adults outside the home [47]. As a result, this period may represent new challenges for young children with CHD and their families [11]. Children with CHD are frequently absent from school and their parents may overprotect them because they may fear that their children will be stigmatized or bullied at school [11, 27, 44, 46]. Therefore, leaving home and attending kindergarten or the first years of elementary school may be a challenge for children with CHD and their parents [11, 48]. In contrast, adolescents and their parents reported a worse perceived physical appearance (e.g., *I feel I am not good looking, I don’t like other people to see my scars, I am embarrassed when others see my body*) than children in the other groups. Adolescents with CHD not only have to face the challenges of living with a chronic condition but also need to accomplish the normative tasks of their developmental period, such as their body image development, which contributes in a fundamental way to the identity of adolescents. Due to their susceptibility to external influence and cultural values [49], adolescents are at high risk of developing a negative body image, which, in turn, can negatively influence their QoL [50]. For these reasons, we may speculate that adolescents with CHD are at higher risk of developing a negative body image than their healthy peers due to physical limitations and scars related to surgical intervention and to their chronic illness in general. In addition, mothers and fathers of our adolescent patients reported lower perceived treatment adherence. During adolescence, the responsibility for managing a chronic disease should gradually shift from the parents to the adolescents themselves [51]; however, the results from previous studies showed that adherence diminished when management of the medical treatment shifts from the parents to the adolescent. According to the literature, several factors such as deductive thinking, independence, enhancement of self-efficacy and parental involvement could impact adherence to treatment in adolescence [51]. However, to date, no studies have previously identified potential risk and protective factors for CHD Italian paediatric patients’ adherence to treatment. Therefore, future studies are needed to corroborate our findings.

Our results also showed that significant differences emerged between UVH patients and other patients when clinical factors were considered. Previous studies have shown a correlation between QoL and the severity of CHD. For example, Marino and colleagues [26], using the Pediatric Cardiac Quality of Life Inventory, found that different CHD population groups obtained a particular QoL score range based on the disease severity and the medical, catheter-based, and surgical therapy required. This correlation between QoL and the severity of CHD seems to be particularly true with regard to the physical dimension of QoL [11, 18, 19, 40, 48, 52, 53]. Indeed, children with a CHD may suffer from an impaired physical capacity (e.g., unpleasant feeling of dyspnoea, related to muscular deconditioning or restrictive lung function [54]); thus, according to these studies, we may speculate that UVH patients reported lower levels of QoL on physical dimensions due to their clinical history (they have undergone more operations during their life—at least three surgical steps—and are forced to take drugs lifetime). In addition to this, results from our study showed that patients who both are taking medications at present and underwent surgery, and their parents, perceived more cardiac symptoms; in contrast, patients who had been operated on only and their parents reported a worse perceived physical appearance. Even in this case, these results are in line with the literature that showed that undergoing surgery and/or tanking cardiac medications resulted in a lower level of QoL in the physical dimensions [55]. Moreover, our study revealed that patients who underwent surgery reported a lower total score on the generic PedsQL and lowered school functioning levels (*e.g., I miss school because of not feeling well, I miss school to go to the doctor or hospital*) than children who take medications only. These results should be interpreted with caution due to the small sample of the Medications-only group (n = 24). However, we may speculate that CHD patients who underwent surgery may have reported lower levels of school functioning because of the frequent absence from school related to medical examinations and/or hospitalizations.

It is important to mention that the results from our study showed that beyond these differences based on the CHD diagnosis, interventions and medications, our patients and their parents reported high levels of QoL, above clinical cut-offs [24, 39]. These results are in line with other studies that revealed how the severity of CHD, in terms of the clinical symptoms and the number of surgical procedures or health interventions, seemed to have a marginal effect on CHD patients’ QoL [56], and that paediatric CHD patients reported lower levels of QoL in the physical domain regardless of the surgery intervention [12]. According to the literature, the combination of medical and social stress seems to have the strongest negative impact on the quality of life in diseased children or adolescents, regardless of its severity [57]. Therefore, we may speculate that a family with sufficient resources, such as higher levels of social support [58], can cope better and restrain the adverse effects on the quality of life, even with a severe disease condition.

### Agreement and directional disagreement between child/adolescent and parent reports on generic and cardiac-specific QoL

Pediatric patient self-report should be considered the standard for measuring patients' QoL. However, there are situations in which parent proxy-reports are needed [24, 25, 59]. Moreover, according to the literature, parental reports could provide important complementary information about children’s QoL [29]. For example, previous studies have shown that parents can report physical and medical aspects in more detail, especially compared with very young children. In contrast, children and adolescents can report detailed information about social exclusion or inclusion [36]. Previous studies reported mixed results, showing agreement between both parental and patient reports and parental overrating of patients’ problems [10]. Overall, our results showed higher percentages of agreement than of disagreement both on generic and condition-specific QoL between mothers and fathers and between parents and children/adolescents. According to Patel and colleagues, a possible explanation for agreement between parental and patient reports could reflect medical improvements in the screening and care of CHD in recent decades. As surgical techniques improve, parents observe fewer differences between their children with CHD and their healthy peers (especially in the physical domains), leading to higher assessments of their quality of life [23] and reducing the degree of disagreement in self-report questionnaires.

Nevertheless, our results revealed a high disagreement rate between parents and patients aged between 5 and 7 years. Specifically, we observed that mothers underrated their children’s anxiety symptoms related to treatment for condition-specific QoL. Moreover, children reported higher scores on cognitive problems than both their mothers and their fathers, and a low agreement rate (23%) was found between patients and fathers in communicative skills. With regard to generic QoL both mothers and fathers showed a trend of underrating their 5–7-years-old children’s problems with generic physical, emotional, social and school functioning. According to previous studies, psychosocial domains related to thoughts and feelings (e.g., anxiety symptoms) are often more difficult for parents to discern through observation, increasing the potential for discrepancies [15, 24, 27, 28, 60]. Moreover, as discussed above, chronic illness, such as CHD, could impact the development of socioemotional, problem-solving and interaction skills during this age range, thereby limiting the ability of young children to identify and express their concerns to parents [57]. However, according to previous studies on CHD patients, the differences in QoL perception might also be due to the different expectations regarding the patients’ social, cognitive and intellectual abilities between parents and the patients themselves [10, 11], especially in the case in which parents underrate their children’s problems. Our results, especially on generic QoL, are in line with those of Uzark and colleagues [15] who showed that children with mild cardiovascular disease (CVD) reported poorer psychosocial QoL than those perceived by their parents and with other studies [56]. According to Hemingsson et al. [61], these results may reflect that in domains where children report more difficulties or lower QoL than parents, children’s opinions may not always be elicited in pediatric settings. Instead, their parents are the primary informants.

For the first time, our study has explored agreement and directional disagreement between Italian CHD pediatric patients and their parents, both mothers and fathers, identifying possible conditions under which parent proxy-report instruments achieve better agreement with child self-report instruments that could facilitate clinicians’ interpretation of QoL outcomes. Including caregivers’ assessment can be an essential aspect in evaluating a child with CHD from the perspective of the use of healthcare facilities and the quality of communication between patients and their caregivers [7, 10]. In addition, our study highlighted the importance of including both mothers and fathers in the QoL proxy-report evaluations because they could perceive their children’s QoL dimensions differently. In our sample of children aged 5–7 years, even though mothers and fathers both generally agreed with their children, mothers showed higher disagreement rates on anxiety symptoms while fathers showed higher disagreement rates on communicative skills. According to the literature, mothers are most often the primary caregivers, and they report higher distress and lower QoL, factors that can influence proxy ratings compared to fathers [62]. However, to date, no studies have previously explored agreement and directional disagreement in Italian CHD patients with both fathers and mothers. Thus, future studies are needed to corroborate our findings.

The present study results could increase the use of patient-related outcomes in clinical practice and guide clinicians’ efforts aimed at prevention and intervention. Moreover, they could enable clinicians and researchers to broaden their understanding of CHD pediatric patients’ well-being [31] by providing a comprehensive understanding of CHD pediatric patients’ QoL due to the inclusion of both patients’ and parents’ perspectives during clinical assessments.

### Limitations

Our study is not without limitations. First, it does not include a control group of healthy children. In future studies a multicenter approach should be taken into account in order to corroborate our findings. Second, it does not evaluate the role of potential risk and protective factors related to the QoL of CHD pediatric patients. Future studies should perform multivariate analyses, including as covariates patients’ age, type of CHD diagnosis, other clinical factors (e.g., number of surgeries, comorbidities, time since last surgery) and patients’ and parental psychological wellbeing. Another limitation is the cross-sectional research design. Follow-up measurements of patients’ QoL are needed to monitor patients’ QoL and familiar adjustment longitudinally. Finally, our study does not include an evaluation of QoL of CHD patients with neurodevelopmental disabilities and/or intellectual disabilities, and parental mental health.

## Conclusions

QoL levels in Italian children and adolescents with CHD were found to be high, above the clinical cut-offs [24, 39], according to both the patient reports and the parent-reports. Our study contributed to the growing body of knowledge on QoL in CHD which emphasizes the need for these families to receive support from multidisciplinary standardized care, including psychological consultations and support. Specifically, our results could be of importance to clinical practice and future research because they highlighted the importance of including QoL assessment in routine clinical practice, especially for 5-to7-year-old patients, during the transition from home to preschool or to primary school, and for adolescent patients, to support their body image development and adherence to the medical treatment process. Our results and findings from previous studies suggest that it is necessary for health care providers to monitor treatment decisions in the context of the social, emotional and cognitive expectations of children themselves and consider these factors in the clinical decision- making process. Moreover, our results highlight the importance of broadening the perspective on pediatric CHD patients, including both patients and parents in QoL assessment. Indeed, although the parent–child-agreement rate was mostly high, some discrepancies occurred, highlighting the need for a multi-informant approach to obtain reliable information. Due to multiple sources of information (patients, mothers and fathers), this study allows clinicians to have a broader view of the patient’s QoL. This information can be used as a tool to help parents better understand the child's real needs, thereby reducing over- or underestimating the child's quality of life. This, in turn, would therefore avoid excessive overprotection or, on the contrary, normalization of the symptoms or the patients’ clinical condition.

## Supplementary Information

**Additional file 1**. **Supplementary Table 1.** Differences between patients who underwent surgery interventions and/or are taking cardiac medications.

**Additional file 2**. **Supplementary Fig. 1.** Agreement and directional disagreement between mothers and fathers of 2-4 y. patients on PedsQL Cardio. **Supplementary Fig. 2.** Agreement and directional disagreement between mothers and fathers of 2-4 y. patients on PedsQL General. **Supplementary Fig. 3.** Agreement and directional disagreement between mothers and fathers of 5-7 y. patients on PedsQL Cardio. **Supplementary Fig. 4.** Agreement and directional disagreement between mothers and fathers of 5-7 y. patients on PedsQL Generic. **Supplementary Fig. 5.** Agreement and directional disagreement between mothers and fathers of 8-12 y. patients on PedsQL Cardio. **Supplementary Fig. 6.** Agreement and directional disagreement between mothers and fathers of 8-12 y. patients on PedsQL Generic. **Supplementary Fig. 7.** Agreement and directional disagreement between mothers and fathers of 13-18 y. patients on PedsQL Cardio. **Supplementary Fig. 8.** Agreement and directional disagreement between mothers and fathers of 13-18 y. patients on PedsQL Generic

**Additional file 3**.** Supplementary Fig. 9.** Agreement and directional disagreement between 5-7 y. patients’ and parents’ reports on PedsQL Cardio. **Supplementary Fig. 10.** Agreement and directional disagreement between 5-7 y. patients’ and parents’ reports on PedsQL Generic. **Supplementary Fig. 11.** Agreement and directional disagreement between 8-12 y. patients’ and parents’ reports on PedsQL Cardio. **Supplementary Fig. 12** Agreement and directional disagreement between 8-12 y. patients’ and parents’ reports on PedsQL Generic. **Supplementary Fig. 13.**Agreement and directional disagreement between 13-18 y. patients’ and parents’ reports on PedsQL Cardio. **Supplementary Fig. 14.** Agreement and directional disagreement between 13-18 y. patients’ and parents’ reports on PedsQL Generic.

## Data Availability

Data are stored in Hospital database (and available from the corresponding author on reasonable request).

## Abbreviations

Ao: Aortopathies
CHD: Congenital heart disease
ECG: Electrocardiography
MID: Minimally important differences
PedsQL: Paediatric quality of life
QoL: Quality of life
RV-PA conduit: Right ventricular-pulmonary artery conduit
TGA: Transposition to the Great Artery
ToF: Tetralogy of Fallot
UVH: Univentricular heart
