# Supplementary material for: Health-related quality of life in Italian children and adolescents with congenital heart diseases
Source: BMC Cardiovasc Disord. 2022 Apr 15;22:173. doi: 10.1186/s12872-022-02611-y (PMC9013137; doi:10.1186/s12872-022-02611-y)
Supplement: Supplementary file 1 — Additional file 1. Supplementary Table 1. Differences between patients who underwent surgery interventions and/or are taking cardiac medications. [file 12872_2022_2611_MOESM1_ESM.pdf]

Supplementary Table 1. Differences between patients who underwent surgery interventions and/or are taking cardiac medications

	No surgery or Medications	Surgery	Medications	Surgery + Medications	***P***(Kruskal -Wallis)	**Significant Comparisons**
	**Median (IQR)**	**Median (IQR)**	**Median (IQR)**	**Median (IQR)**		
**Patient Report**						
Peds Cardio	(n=49)	(n=247)	(n=24)	(n=131)		
1.Cardiac symptoms	**86 (18)**	78 (15)	84 (21)	**78 (18)**	**.014**	sur+med < no sur/med
2.Adherence to treatment	/NA	/NA	92.5 (15)	95 (10)	.246	
3.Perceived Physical appearance	100 (35.5)	92 (25)	**100 (9.5)**	**83 (33)**	**.012**	sur+med < sur
4.Anxiety towards treatment	75 (41)	87 (38)	81 (48.5)	87 (31)	.074	
5.Cognitive status	80 (27,5)	80 (27.5)	80 (15)	80 (25)	.534	
6.Communicative skills	75 (33.5)	83 (33)	79 (33)	83 (33)	.286	
7.Cardio Total	78 (17)	79 (15)	83 (15)	82 (17)	.130	
Peds Generic	(n=49)	(n=246)	(n=24)	(n=131)		
1.Physical Functioning	84 (19)	81 (22)	87 (15.25)	78 (21)	.060	
2.Emotional Functioning	75 (25)	75 (25)	82.5 (18,75)	80 (30)	.055	
3.Social Functioning	90 (30)	90 (25)	87.5 (18.75)	90 (20)	.950	
4.School Functioning	80 (20)	**80 (25)**	**90 (11.25)**	80 (20)	.050	Sur < med
5.Generic Total	83 (21.5)	**78 (15.25)**	**87.5 (12.5)**	79 (22)	.050	Sur < med
**Mother Report**						
Peds Cardio	(n=49)	(n=243)	(n=26)	(n=110)		
1.Cardiac symptoms	**86 (23)**	**82 (18)**	78 (22)	**78 (22)**	**.009**	Sur+med < no sur/med<br>Sur+med < sur
2.Adherence to treatment	/NA	/NA	95 (21.25)	95 (10)	.304	
3.Perceived Physical appearance	100 (25)	83 (33)	100 (32.25)	79 (50)	.060	
4.Anxiety towards treatment	75 (40.5)	69 (44)	75 (63)	69 (58.5)	.825	
5.Cognitive status	65 (37.5)	60 (35)	62.5 (26.25)	70 (36.25)	.060	
6.Communicative skills	83 (39.75)	83 (42)	71 (42)	83 (44)	.921	
7.Cardio Total	76 (22.5)	73 (18)	77.5 (21.25)	76.5 (20.25)	.424	
Peds Generic	(n=49)	(n=247)	(n=25)	(n=112)		
1.Physical Functioning	91 (26.5)	**91 (22)**	87 (29.5)	**84 (35)**	**.014**	Sur+med < sur
2.Emotional Functioning	75 (30)	75 (30)	70 (27.5)	75 (30)	.921	
3.Social Functioning	90 (20)	90 (25)	95 (30)	87.5 (35)	.293	
4.School Functioning	90 (37.5)	80 (28.5)	85 (36.25)	80 (35)	.514	

5.Generic Total	87<br>(20)	83<br>(18)	79<br>(23.5)	80<br>(30.75)	.166	
**Father Report**						
Peds Cardio	(n=28)	(n=188)	(n=15)	(n=91)		
1.Cardiac symptoms	86<br>(13.25)	86<br>(18)	82<br>(28)	78<br>(21)	.060	
2.Adherence to treatment	/NA	/NA	95<br>(17)	97.5<br>(10)	.527	
3.Perceived Physical appearance	**100**<br>**(0)**	**92**<br>**(25)**	100<br>(13)	**83**<br>**(33)**	**.000**	Sur+med < no sur/med<br>Sur+med < sur
4.Anxiety towards treatment	87<br>(48.5)	75<br>(48.5)	69<br>(63)	62<br>(57)	.159	
5.Cognitive status	60<br>(55)	70<br>(30)	75<br>(30)	70<br>(40)	.099	
6.Communicative skills	75<br>(50)	83<br>(33)	100<br>(42)	83<br>(42)	.095	
7.Cardio Total	76.5<br>(30.25)	77<br>(17.5)	76<br>(24)	78<br>(20)	.934	
Peds Generic	(n=28)	(n=188)	(n=15)	(n=92)		
1.Physical Functioning	92.5<br>(16)	91<br>(19)	94<br>(31)	85.5<br>(24.25)	.062	
2.Emotional Functioning	80<br>(20)	80<br>(20)	65<br>(35)	75<br>(30)	.243	
3.Social Functioning	95<br>(20)	90<br>(20)	90<br>(25)	90<br>(30)	.283	
4.School Functioning	93.5<br>(23.75)	80<br>(25)	80<br>(20)	85<br>(30)	.306	
5.Generic Total	88.5<br>(13.75)	86<br>(17)	78<br>(28)	84<br>(28.75)	.230	

Note. IQR=Interquartile Range; med=medications; n=no. of patients; sur=surgery
