# Supplementary material for: Health-related quality of life in Italian children and adolescents with congenital heart diseases
Source: BMC Cardiovasc Disord. 2022 Apr 15;22:173. doi: 10.1186/s12872-022-02611-y (PMC9013137; doi:10.1186/s12872-022-02611-y)

Supplementary Fig. 1 Agreement and directional disagreement between mothers and fathers of 2-4 y. patients on PedsQL Cardio

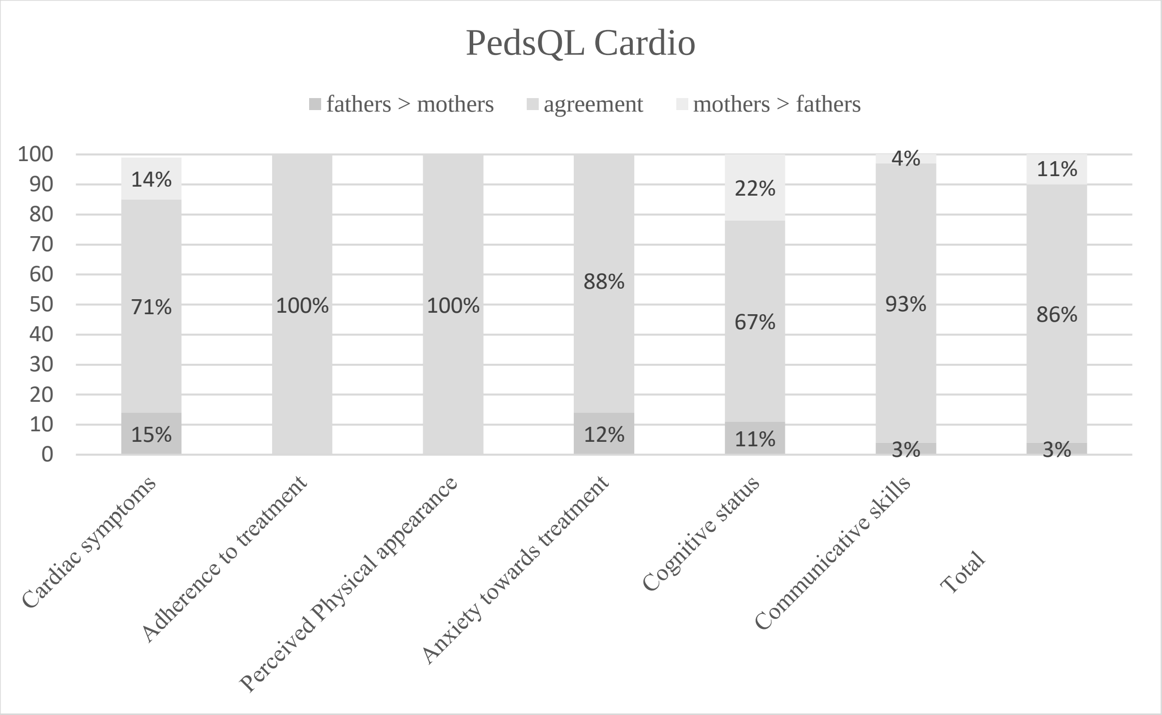

Supplementary Fig. 2 Agreement and directional disagreement between mothers and fathers of 2-4 y. patients on PedsQL General

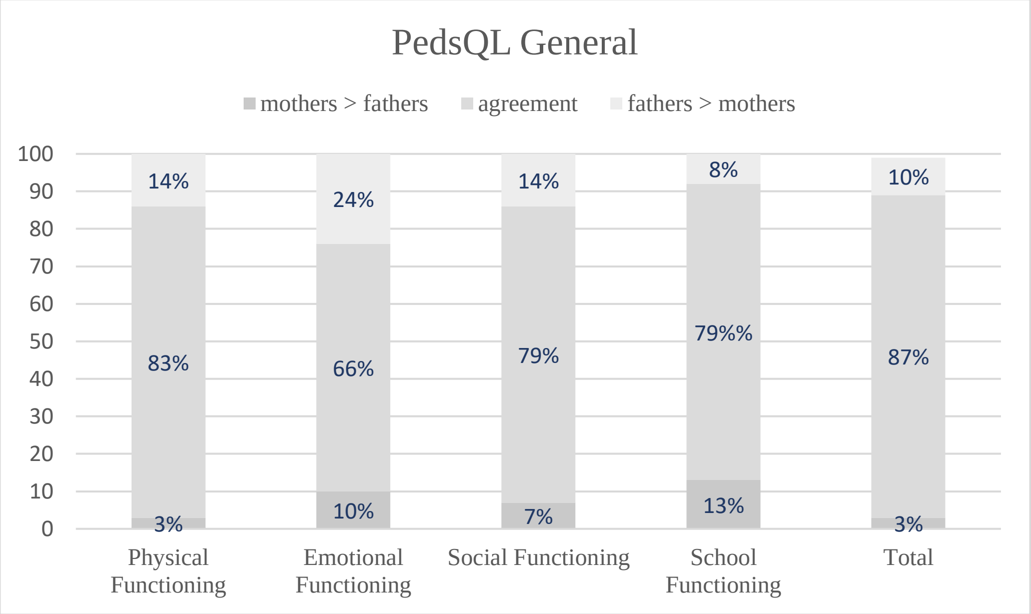

Supplementary Fig. 3 Agreement and directional disagreement between mothers and fathers of 5-7 y. patients on PedsQL Cardio

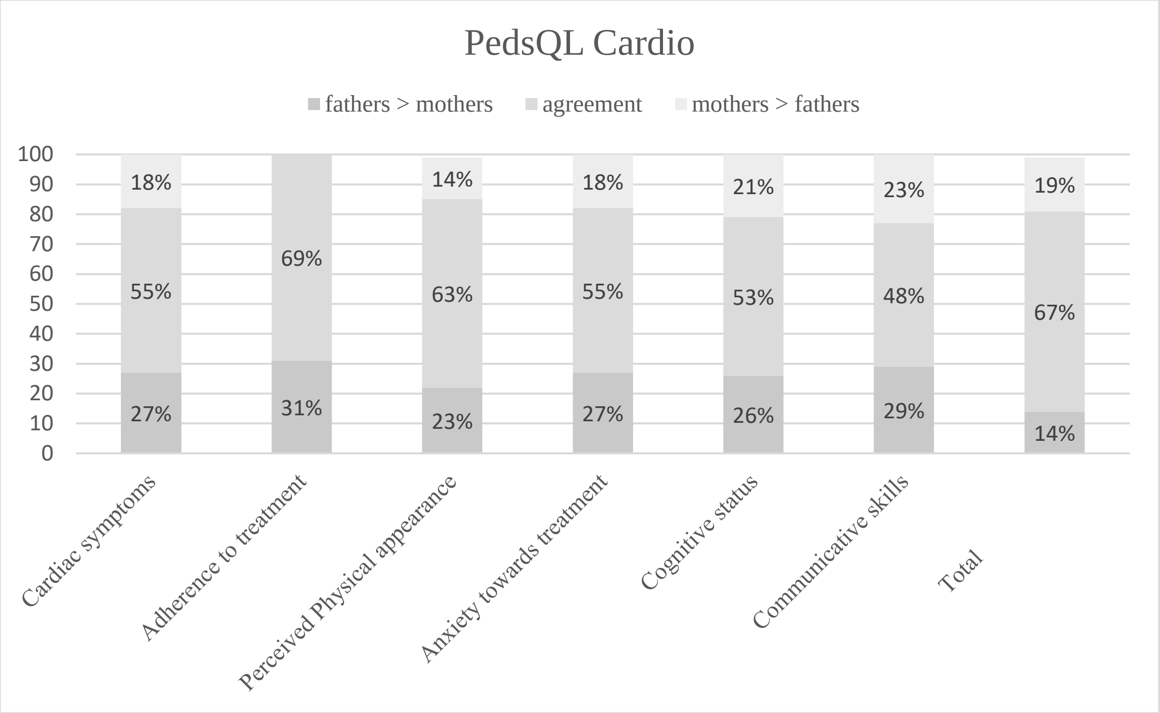

Supplementary Fig. 4 Agreement and directional disagreement between mothers and fathers of 5-7 y. patients on PedsQL Generic

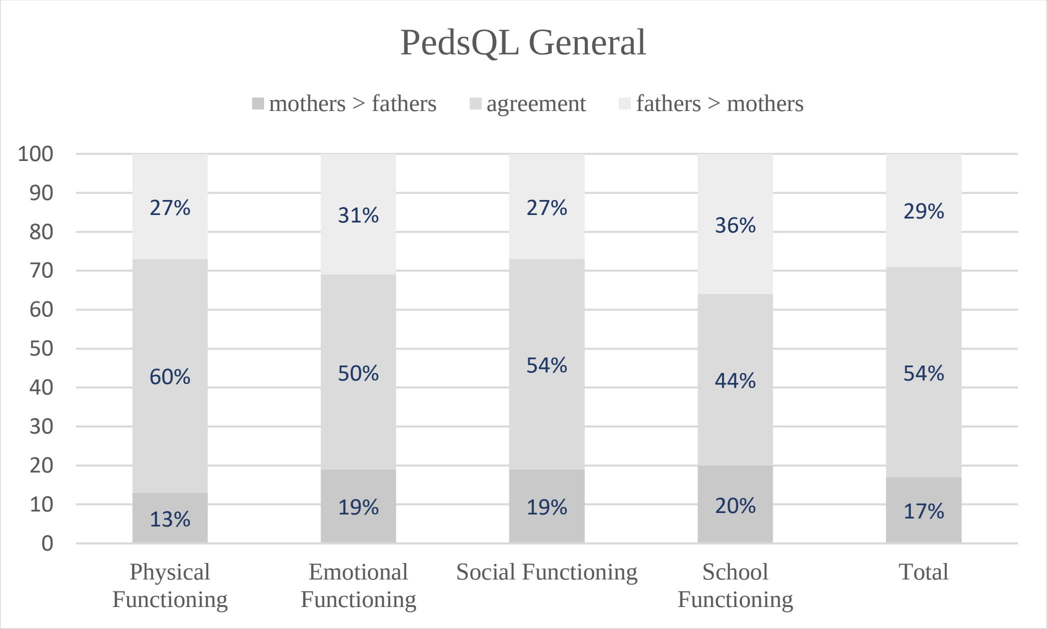

Supplementary Fig. 5 Agreement and directional disagreement between mothers and fathers of 8-12 y. patients on PedsQL Cardio

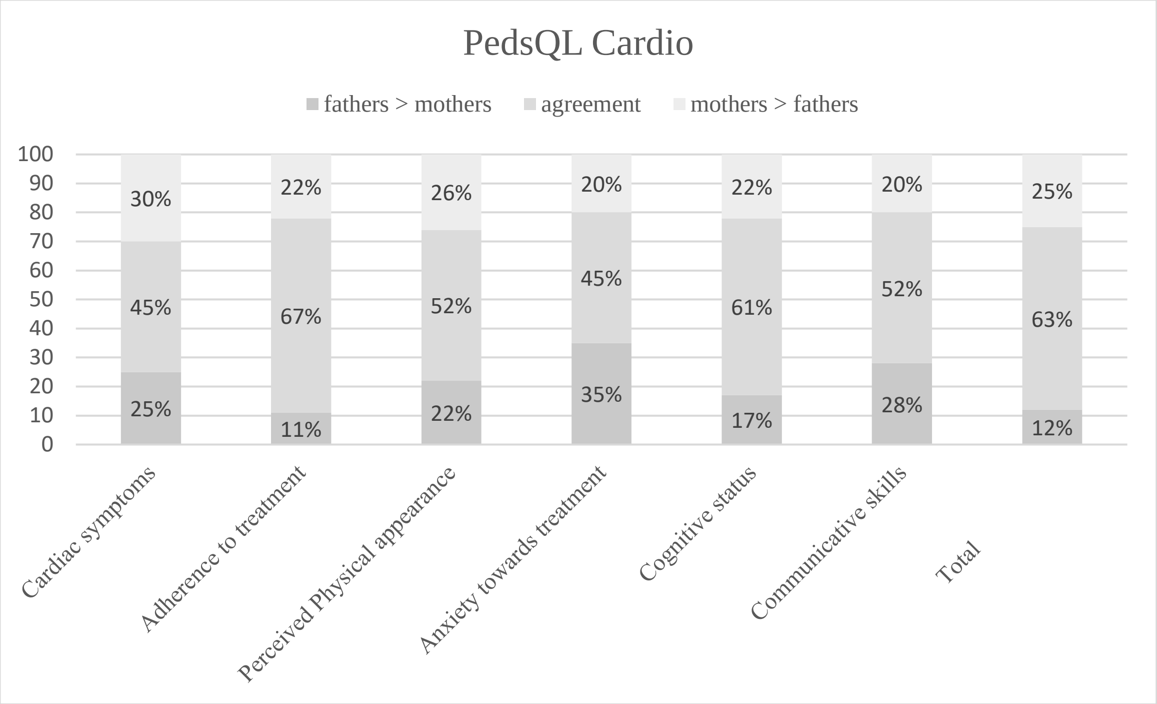

Supplementary Fig. 6 Agreement and directional disagreement between mothers and fathers of 8-12 y. patients on PedsQL Generic

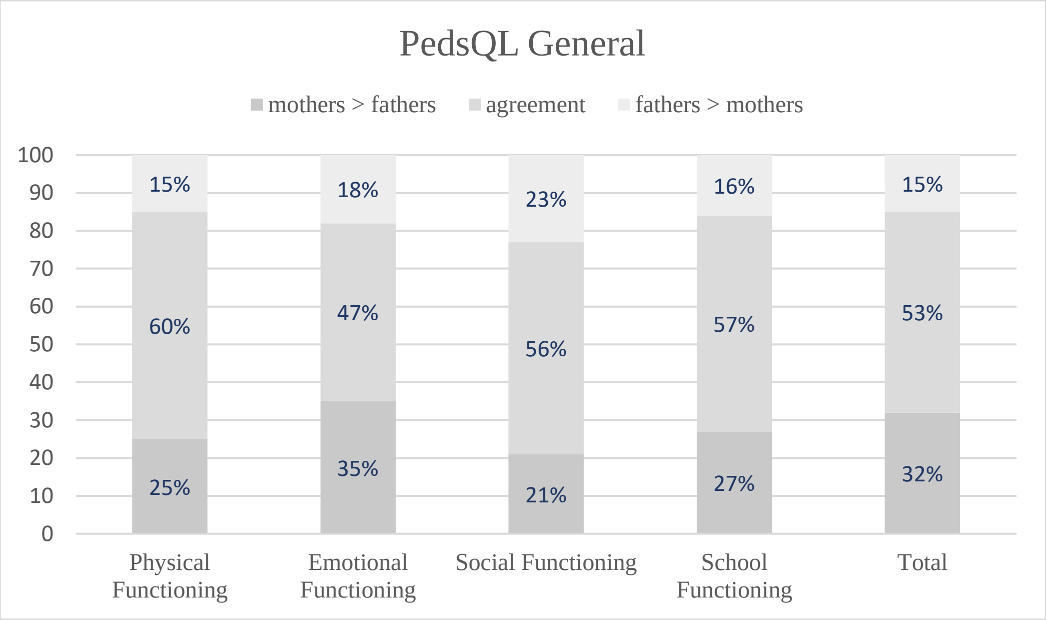

Supplementary Fig. 7 Agreement and directional disagreement between mothers and fathers of 13-18 y. patients on PedsQL Cardio

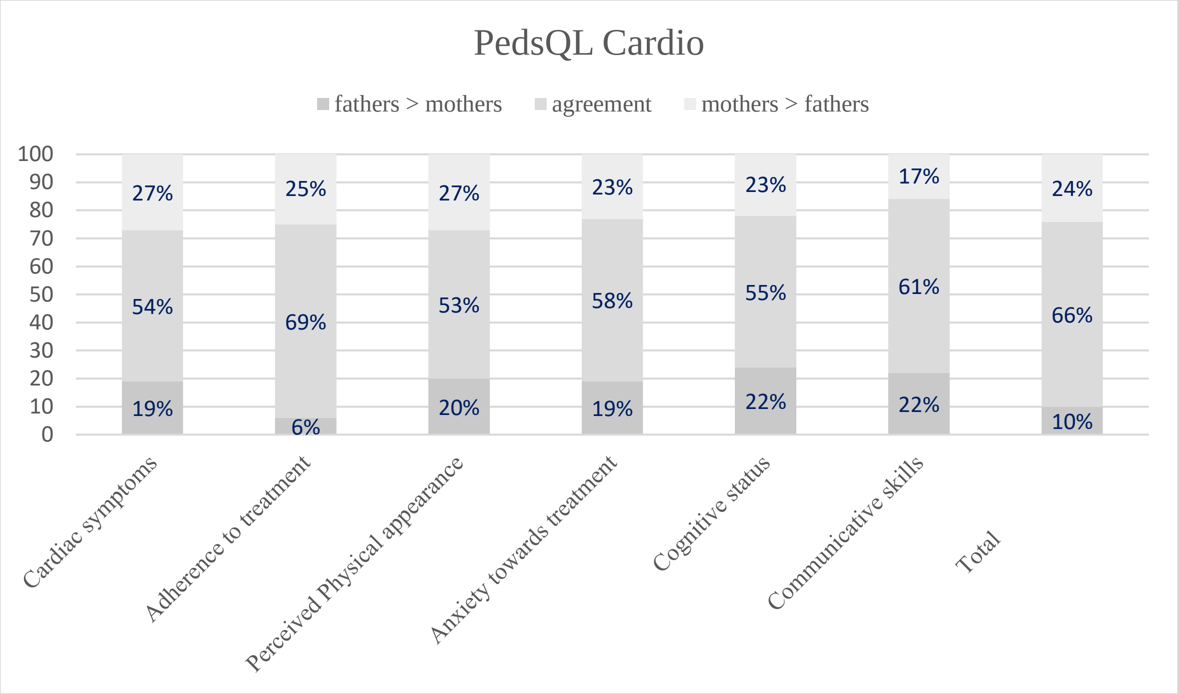

Supplementary Fig. 8 Agreement and directional disagreement between mothers and fathers of 13-18 y. patients on PedsQL Generic

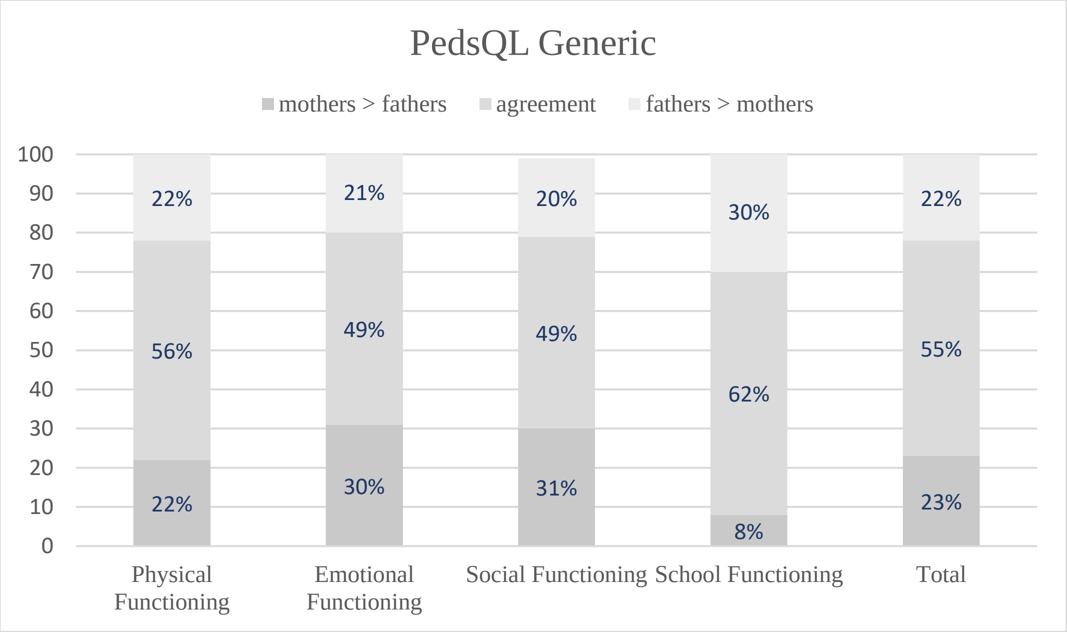

Supplement: Supplementary file 2 — Additional file 2. Supplementary Fig. 1. Agreement and directional disagreement between mothers and fathers of 2-4 y. patients on PedsQL Cardio. Supplementary Fig. 2. Agreement and directional disagreement between mothers and fathers of 2-4 y. patients on PedsQL General. Supplementary Fig. 3. Agreement and directional disagreement between mothers and fathers of 5-7 y. patients on PedsQL Cardio. Supplementary Fig. 4. Agreement and directional disagreement between mothers and fathers of 5-7 y. patients on PedsQL Generic. Supplementary Fig. 5. Agreement and directional disagreement between mothers and fathers of 8-12 y. patients on PedsQL Cardio. Supplementary Fig. 6. Agreement and directional disagreement between mothers and fathers of 8-12 y. patients on PedsQL Generic. Supplementary Fig. 7. Agreement and directional disagreement between mothers and fathers of 13-18 y. patients on PedsQL Cardio. Supplementary Fig. 8. Agreement and directional disagreement between mothers and fathers of 13-18 y. patients on PedsQL Generic [file 12872_2022_2611_MOESM2_ESM.pdf]
