# Supplementary material for: Health-related quality of life in Italian children and adolescents with congenital heart diseases
Source: BMC Cardiovasc Disord. 2022 Apr 15;22:173. doi: 10.1186/s12872-022-02611-y (PMC9013137; doi:10.1186/s12872-022-02611-y)

Supplementary Fig. 9 Agreement and directional disagreement between 5-7 y. patients' and parents' reports on PedsQL Cardio

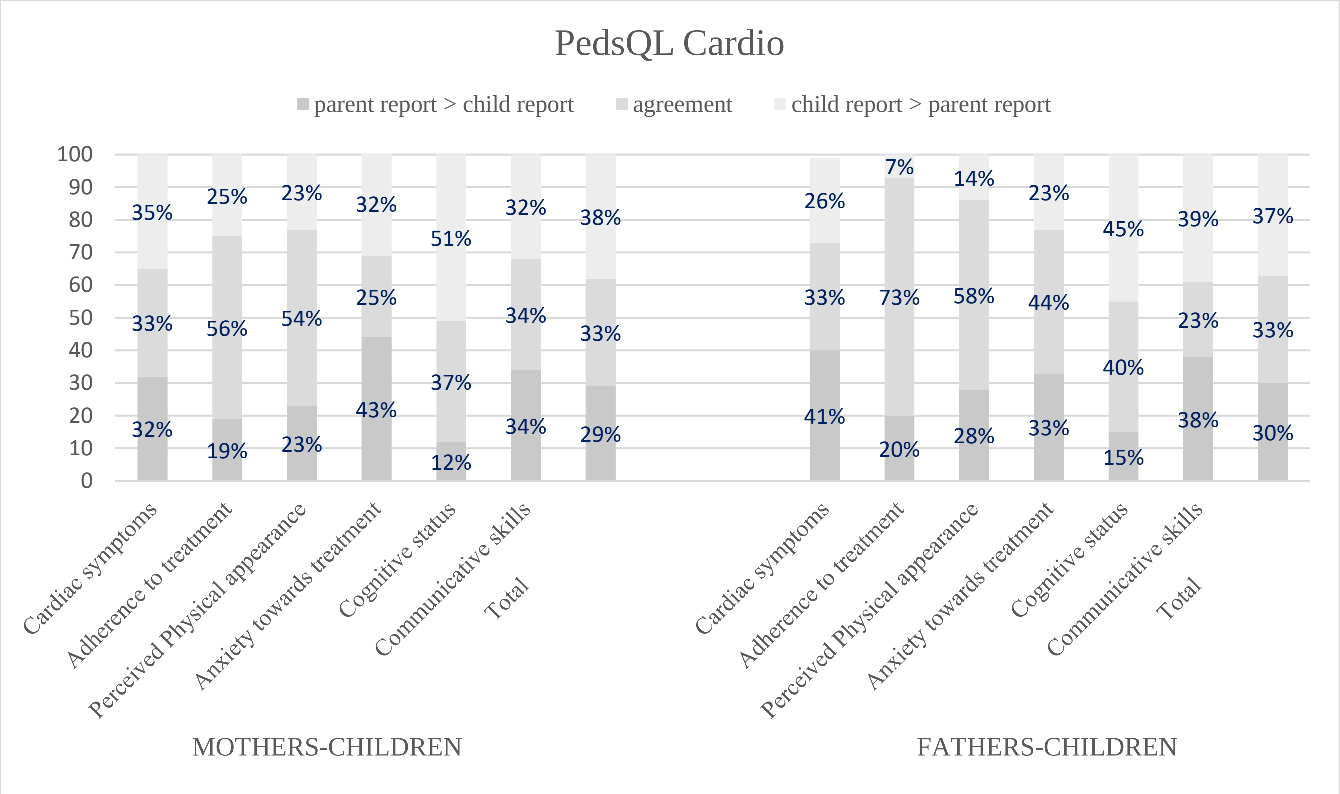

Supplementary Fig. 10 Agreement and directional disagreement between 5-7 y. patients' and parents' reports on PedsQL Generic

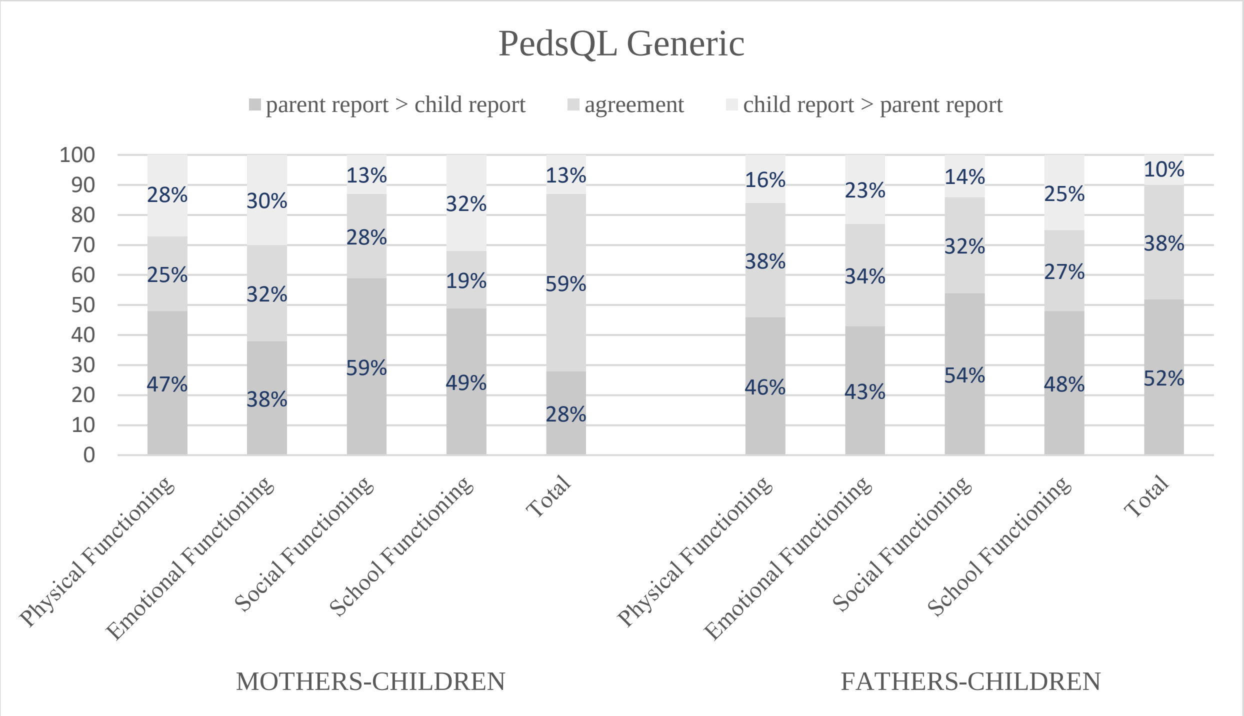

Supplementary Fig. 11 Agreement and directional disagreement between 8-12 y. patients' and parents' reports on PedsQL Cardio

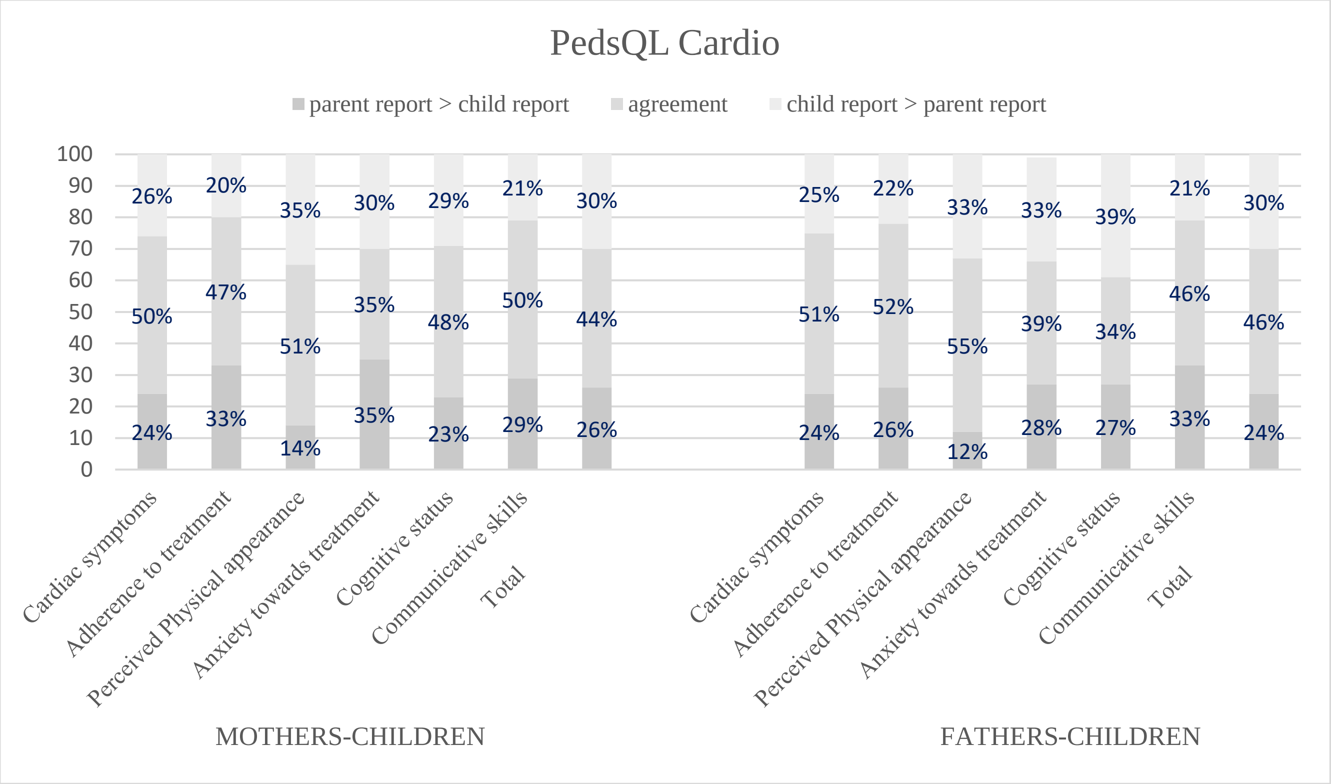

Supplementary Fig. 12 Agreement and directional disagreement between 8-12 y. patients' and parents' reports on PedsQL Generic

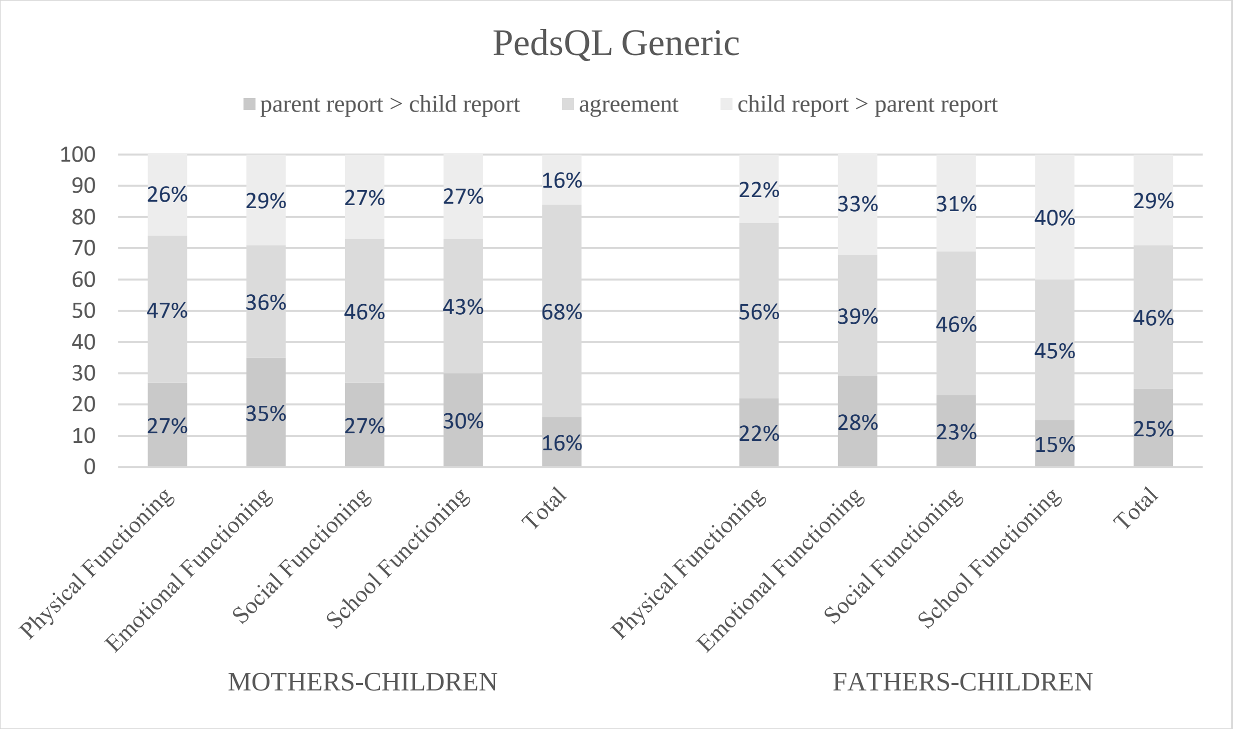

Supplementary Fig. 13 Agreement and directional disagreement between 13-18 y. patients' and parents' reports on PedsQL Cardio

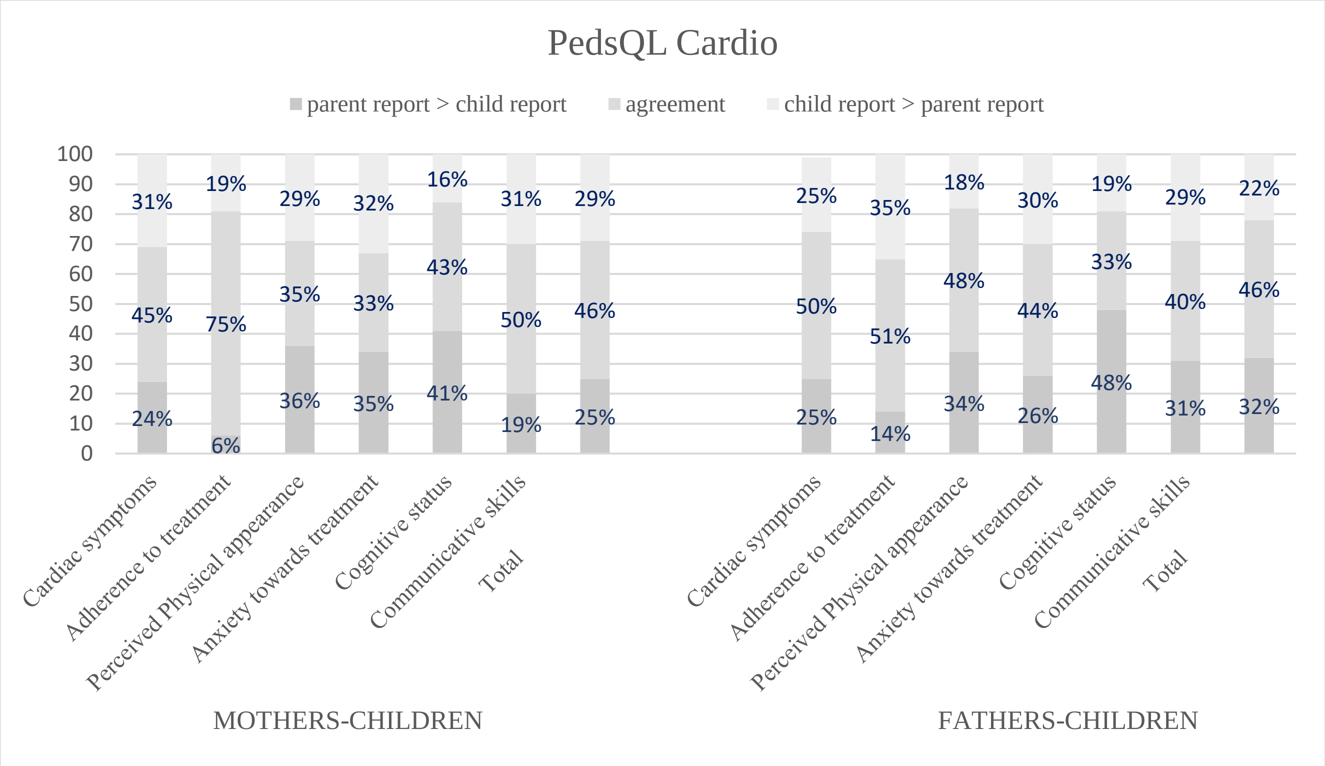

Supplementary Fig. 14 Agreement and directional disagreement between 13-18 y. patients' and parents' reports on

PedsQL Generic

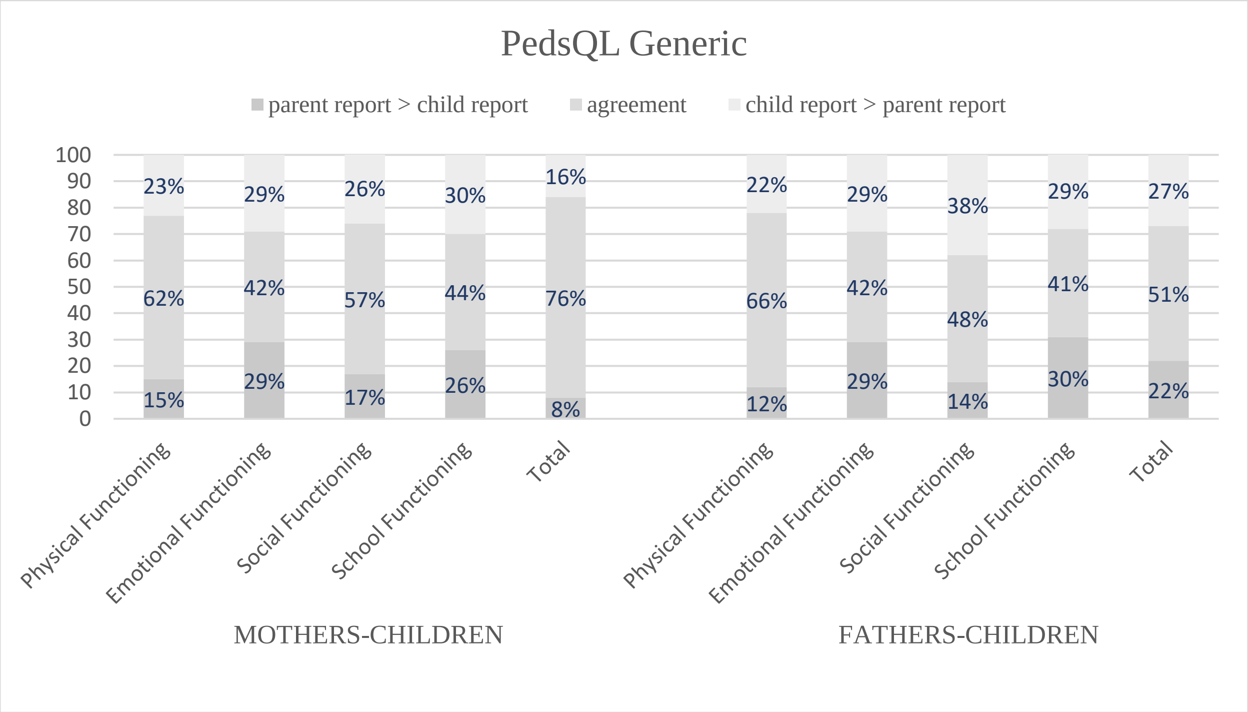

Supplement: Supplementary file 3 — Additional file 3. Supplementary Fig. 9. Agreement and directional disagreement between 5-7 y. patients’ and parents’ reports on PedsQL Cardio. Supplementary Fig. 10. Agreement and directional disagreement between 5-7 y. patients’ and parents’ reports on PedsQL Generic. Supplementary Fig. 11. Agreement and directional disagreement between 8-12 y. patients’ and parents’ reports on PedsQL Cardio. Supplementary Fig. 12 Agreement and directional disagreement between 8-12 y. patients’ and parents’ reports on PedsQL Generic. Supplementary Fig. 13.Agreement and directional disagreement between 13-18 y. patients’ and parents’ reports on PedsQL Cardio. Supplementary Fig. 14. Agreement and directional disagreement between 13-18 y. patients’ and parents’ reports on PedsQL Generic. [file 12872_2022_2611_MOESM3_ESM.pdf]
